# Structure-Activity Relationship of Dialkoxychalcones to Combat Fish Pathogen *Saprolegnia australis*

**DOI:** 10.3390/molecules23061377

**Published:** 2018-06-07

**Authors:** Iván Montenegro, Ociel Muñoz, Joan Villena, Enrique Werner, Marco Mellado, Ingrid Ramírez, Nelson Caro, Susana Flores, Alejandro Madrid

**Affiliations:** 1 Escuela de Obstetricia y Puericultura, Facultad de medicina, Campus de la Salud, Universidad de Valparaíso, Angamos 655, Reñaca, Viña del Mar 2520000, Chile; ivan.montenegro@uv.cl; 2 Institute of Food Science and Technology, University Austral of Chile, Valdivia 5090000, Chile; ocielmunoz@uach.cl; 3 Centro de Investigaciones Biomédicas (CIB), Escuela de Medicina, Universidad de Valparaíso, Av. Hontaneda N° 2664, Valparaíso 2340000, Chile; juan.villena@uv.cl; 4 Departamento De Ciencias Básicas, Campus Fernando May Universidad del Biobío, Avda. Andrés Bello s/n casilla 447, Chillán 3780000, Chile; ewerner@ubiobio.cl; 5 Instituto de Química, Facultad de Ciencias, Pontificia Universidad Católica de Valparaíso, Av. Universidad #330, Curauma, Valparaíso 2340000, Chile; marco.mellado@pucv.cl; 6 Centro de Biotecnología “Dr. Daniel Alkalay Lowitt”, Universidad Técnica Federico Santa María, Avda. España 1680, Valparaíso 2340000, Chile; ingrid.ramirez@usm.cl; 7 Centro de Investigación Australbiotech, Universidad Santo Tomás, Avda. Ejército 146, Santiago 8320000, Chile; ncaro@australbiotech.cl; 8 Departamento de Química, Facultad de Ciencias Naturales y Exactas, Universidad de Playa Ancha, Avda. Leopoldo Carvallo 270, Playa Ancha, Valparaíso 2340000, Chile; s.flores.gonzalez@gmail.com

**Keywords:** 2′,4′-dihydroxychalcone, chalcones, oomycetes, *Saprolegnia*

## Abstract

To investigate the anti-*Saprolegnia* activities of chalconic compounds, nine dialkoxychalcones **2**–**10**, along with their key building block 2′,4′-dihydroxychalcone **1**, were evaluated for their potential oomycide activities against *Saprolegnia australis* strains. The synthesis afforded a series of *O*-alkylated derivatives with typical chalcone skeletons. Compounds **4**–**10** were reported for the first time. Interestingly, analogue **8** with the new scaffold demonstrated remarkable in vitro growth-inhibitory activities against *Saprolegnia* strains, displaying greater anti-oomycete potency than the standard drugs used in the assay, namely fluconazole and bronopol. In contrast, a dramatic loss of activity was observed for *O*-alkylated derivatives **2**, **3**, **6**, and **7**. These findings have highlighted the therapeutic potential of the natural compound **1** scaffold to be exploitable as a drug lead with specific activity against various *Saprolegnia* strains.

## 1. Introduction

Aquatic oomycetes (water molds) cause problems and economic losses in fish hatcheries worldwide. Oomycete infections, particularly by different species of the genus *Saprolegnia* [1], can also occur as a secondary infection to bacterial or viral infections [2,3]. The management of *Saprolegnia* sp. infections had relied on therapeutic use of malachite green for many decades, until malachite green was banned in the European Union in 2002 [4]. Several studies seeking replacements for malachite green have been performed [5,6,7], but no single effective, economical, and safe substitute has yet been found. Bronopol treatment [8] is currently the most used method against saprolegniasis in fish farms. However, there are growing concerns regarding its use within the industry, since it poses acute toxicity to several fish species and potentially harmful effects to human health [9]. All of these reasons confirm the need for new infection control methods in order to avoid destructive infection and prevent economic losses in salmonid producing countries.

Chalcones, i.e., compounds containing two phenyl rings connected with an alpha-beta unsaturated ketone, have varied and potent biological properties, making them an attractive scaffold for drug discovery [10]. Specifically, chalcones with *O*-substitution are of interest, since, to date, only 350 oxyprenylated derivatives have been isolated and/or synthesized, and these compounds possess a wide range of valuable and promising pharmacological activities [11,12,13]. In this context, some structure-activity relationship studies have suggested that the antimicrobial effect of the synthesized chalcones could be attributable to the presence of a substitution of the chalcone ring system with *O*-alkyl groups, which is thought to increase their lipophilicity and, consequently, enhance their antimicrobial activity through interaction with cellular membranes [14,15]. The role of lipophilicity in drug discovery and design is a crucial one [16]. In line with the search for new anti-oomycete agents, we report here on the synthesis, structure elucidation, and biological effects of a series of dialkoxychalcones **2**–**10** derived from 2′,4′-dihydroxychalcone **1**.

## 2. Results

### 2.1. Synthesis and Characterization

**Scheme 1:**
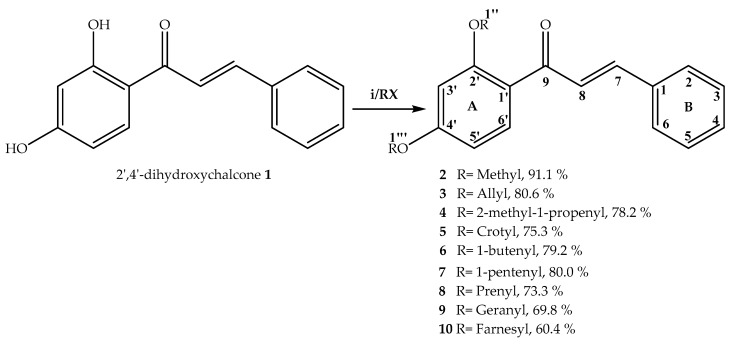
Synthetic route for dialkoxychalcones **2**–**10**. Reagents and conditions: K_2_CO_3_, dry DMF, 4 h at reflux.

The dialkoxychalcones 2–10 were synthesized from 2′,4′-dihydroxychalcone 1 by condensation with the corresponding alkyl bromide in the presence of potassium carbonate with DMF (Scheme 1) [17].

**Figure 1:**
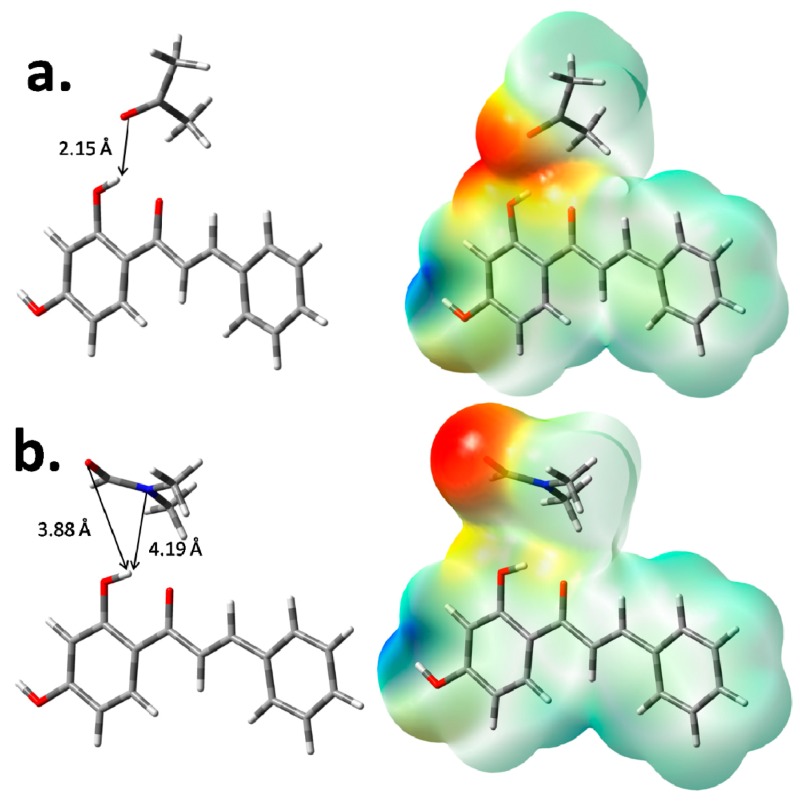
Density functional theory (DFT) results with H-bond and electrostatic potential map (ESP) of **1** with (**a**) acetone and (**b**) DMF.

The use of a polar aprotic solvent, such as DMF in this synthesis, decreased the intensity of the hydrogen bond, allowing the formation of the dialkylated products [18,19]. In our previous work, a synthesis was performed in a similar manner by using alkyl halides and potassium carbonate. In all examples, only monoalkoxychalcones were obtained as a consequence of the use of acetone as a solvent [12,13]. Thus, we used density functional theory (DFT) to calculate the solvent effects of polar aprotic solvents DMF and acetone (Figure 1).

Figure 1a shows the solvent is near the 2′-OH group forming a hydrogen bridge between the oxygen of acetone and the hydrogen substituent with a distance of 2.15 Å. Additionally, the electrostatic potential map (ESP) shows a negative zone on the oxygen of acetone that has an influence on the hydrogen of the 2′-OH group, confirming the hypothesis of hydrogen bonding and selectivity in mono-alkylation of derivatives. In Figure 1b, the solvent is further away from the 2′-OH group than that observed in Figure 1a; the distance between the nitrogen of the DMF and substituent corresponds to 4.19 Å, while the oxygen has a distance of 3.88 Å. Additionally, ESP-plots show a neutral zone near solvent and substituent. For that reason, use of DMF allows for dialkylation.

The structure of dialkoxychalcones **2**–**10** were confirmed by standard spectroscopic techniques. The ^1^H NMR data for the derivatives **2**–**10** confirms that the hydroxyl peaks of compound 1 are no longer present and that there are additional alkoxy peaks depending on the alkyl groups added, confirming the dialkylation. The spectra of these compounds showed two doublets with *J* ~ 15.5 Hz in the ~7.6 ppm range, confirming the existence of the trans double bond in all the analogs. The presence of carbonyl groups was supported by the signals at δ 191.8–190.5 in ^13^C NMR and 1636–1632 cm^−1^ in the IR spectra, respectively.

### 2.2. Anti-Saprolegnia Activity

The dialkoxychalcones **2**–**10** and their precursor compound **1** were evaluated for their anti-oomycete activities against *Saprolegnia australis* strains according to the method described in the literature [12], with some modifications. The obtained minimum inhibitory concentration (MIC), minimum oomyceticidal concentration (MOC), and the percentage of the membrane damage values for the compounds have been summarized in Table 1.

Two subgroups of dialkoxychalcones with different alkyl chains were studied for their structure-activity correlations: compounds **2**, **6** and **7** with a methoxy and unsaturated chain on the one hand, and compounds **3**–**5** and **8**–**10** with a typical allylic chain on the other.

From the results shown in Table 1, natural compound **1** showed higher inhibition than synthetic compounds; these results coincided with those reported by Flores [12]. However, comparing the activities of **1**–**10**, the compound **8** was the most active derivative against *S. australis*, with MOC of 50 µg·mL^−1^. Compound **4** was the second most active derivate, with MOC of 100 µg·mL^−1^. The third in decreasing order was **5** with MOC of 125 µg·mL^−1^. While **2** and **10** had a moderate effect, **3**, **6**, **7**, and **9** had lower activity against this fish pathogen, presenting MOC values of 175, 175, >200, >200, >200, and >200 µg·mL^−1^, respectively (see Table 1).

### 2.3. Structure-Activity Relationship

Several computational methodologies have been used to study the structure-activity relationship [20], mainly to obtain different molecular descriptors, such as topological, steric, and electronic descriptors. The most used methodology for obtaining electronic descriptors is the quantum chemical calculations based on Density Functional Theory (DFT), because it obtains satisfactory results based on accuracy and reliability [21]. In fact, through DFT, it was possible to calculate different electronic descriptors that led to understanding reactivity in organic molecules on several biological targets [22,23].

According to previously mentioned descriptors in the experimental section, the values of these parameters were calculated for dialkoxychalcones under investigation (in gas and solvent phases). Those that elicited a statistical significance of (*p* ≤ 0.05) were correlated with the respective minimum inhibitory concentration (pMIC). The descriptors considered in the gas phase were: HBD^2^, 1/Lowest Orbital Molecular Unoccupied (LUMO^−1^), 1/η, 1/ω, and Log_10_ω. However, when Hansch’s analysis of statistical depuration was completed, only the descriptors LUMO^−1^ and Log_10_ω were able to explain reactivity differences with 99.99% of certain (*p* < 0.0001) (see Equation (1) and Table S1 Supplementary Material). On the other hand, descriptors considered in the solvent phase were: square hydrogen bonding donor (HBD^2^), 1/rotatable bonding (RB^−1^), 1/Highest Occupied Molecular Orbital (HOMO^−1^), the logarithmic difference between LUMO and HOMO (Log_10_LH), the electronic chemical potential (µ), and 1/hardness (η^−1^). However, when Hansch’s analysis of statistical depuration was completed, only the descriptors HOMO^−1^and η^−1^ were able to explain reactivity differences with 99.99% of certain (*p* < 0.0001) (see Equation (2) and Table S2 Supplementary Material). In the QSAR equations, “n” is the number of data points, “r” is the Pearson’s correlation, “r^2^” is the square of the correlation coefficient and represents the goodness of fit, “SD” is the standard deviation of the multivariable model, “F” is a Fischer test, and “q^2^” is the cross-validation (a measure of the quality of the QSAR model).

The first model (Equation (1)) shows that minimum inhibitory concentration is correlated with Lowest Unoccupied Molecular Orbital (LUMO) and global electrophylicity index (ω), where ω is 30-folds more important than LUMO. The ω descriptor is a concept of measure for energy stabilization, such as in cases when systems receive additional electronic charges from underground [24,25]. Therefore, to increase this activity on *S. australis* an electron withdrawing substituent should be provided for stabilization of additional electronic charges.

pMIC_gas_ = 76.4(15.78) − 6.30(1.51) (LUMO)^−1^ + 191.31(43.91) Log_10_ω, (1)

N = 6, r = 0.972, r^2^ = 0.945, SD = 0.154, F = 25.7, q^2^ = 0.942.

On the other hand, the second model (in solvent phase) correlated 1/HOMO and 1/hardness descriptors, when 1/HOMO is 3-fold more important η. The HOMO descriptor is related to Lewis base [26], and the values negatives increase the anti-oomycete activity. Despite the good fit of the QSAR model, the solvent model has less statistical values than the gas model. However, the same trends with the model based in calculus that includes the solvent effect have been reported [27].

pMIC_solv_ = −2.58(12.97) − 41.00(14.65) (HOMO)^−1^ − 12.65(3.90)(η)^−1^, (2)

N = 6, r = 0.939, r^2^ = 0.881, SD = 0.201, F = 11.1, q^2^ = 0.865.

## 3. Materials and Methods

### 3.1. General Experimental Procedures

All reagents and solvents were purchased from commercial sources (Sigma-Aldrich, St. Louis, MO, USA) and used without purification. All reactions were monitored by thin layer chromatography (TLC) on TLC precoated silica gel 60 F254 glass-backed plates (Merck KGaA, Darmstadt, Germany). Flash column chromatography was performed on silica gel (200–300 mesh) (Merck KGaA, Darmstadt, Germany). Melting points were measured on a SMP3 apparatus (Stuart-Scientific, now Merck KGaA, Darmstadt, Germany) and are uncorrected. Refractive index values of the samples used in the experiment were measured using Abbe’s refractometer (Krüss, Hamburg, Germany). IR spectra were recorded as KBr disks in a FT-IR 6700 spectrometer (Nicolet, Thermo Scientific, San Jose, CA, USA) and frequencies are reported in cm^−1^. The ^1^H, ^13^C, 13C DEPT-135, gs 2D HSQC, and gs 2D HMBC spectra were recorded in CDCl_3_ solutions and are referenced to the residual peaks of CHCl_3_ at δ = 7.26 ppm and δ = 77.0 ppm for ^1^H and ^13^C, respectively, on an Avance 400 Digital NMR spectrometer (Bruker, Rheinstetten, Germany) operating at 400.1 MHz for ^1^H and 100.6 MHz for ^13^C. HRMS were recorded in a MAT 95 XL mass spectrometer (Thermo Finnigan, Bremen, Germany). The natural compound 2′,4′-dihydroxychalcone was purified from the resinous exudate of *Adesmia balsamica* as reported previously [12].

### 3.2. General Procedure for the Synthesis of Bisalkoxychalcones ***2**–**10***

Compound **1** (0.42 mmol) was added to a round-bottom flask with different alkyl bromides (0.84 mmol) and anhydrous potassium carbonate (0.232 g; 1.68 mmol) in dry DMF (5 mL), and refluxed for 4 h at 70 °C. After completion of reaction, the mixture was cooled and diluted with water (50 mL) and extracted with ethyl acetate (3 × 20 mL). The combined organic layers were dried over anhydrous Na_2_SO_4_, filtered and concentrated under vacuum. The products were purified by crystallization from methanol, or the crude was dissolved in CH_2_Cl_2_ (5 mL) and chromatographed on silica gel with hexane/ethyl acetate mixtures of increasing polarity.

*(2E)-1-(2,4-Dimethoxyphenyl)-3-phenylprop-2-en-1-one* (**2**). Yellow oil. Yield: 91.1%. ^1^H NMR (400.1 MHz, CDCl_3_): 7.76 (m, 1H, H-6′); 7.69 (d, *J* = 15.6 Hz, 1H, H-7); 7.62 (m, 2H, H-2 and H-6); 7.52 (1H, *J* = 15.5 Hz, H-8); 7.43 (m, 3H, H-3, H-4 and H-5); 6.55 (m, 2H, H-3′ and H-5′); 3.88 (s, 3H, H-1″); 3.84 (s, 3H, H-1′′′). Spectroscopic data and physical properties of compound **2** were consistent with those reported in the literature [28].

*(2E)-1-[2,4-Bis(allyloxy)phenyl]-3-phenylprop-2-en-1-one* (**3**). Yellow oil. Yield: 80.6%. ^1^H NMR (400.1 MHz, CDCl_3_): 7.77 (m, 1H, H-6′); 7.69 (d, *J* = 15.6 Hz, 1H, H-7); 7.61 (m, 2H, H-2 and H-6); 7.52 (1H, *J* = 15.5 Hz, H-8); 7.43 (m, 3H, H-3, H-4 and H-5); 6.53 (m, 2H, H-3′ and H-5′); 6.06 (m, 2H, H-2″ and H-2′′′); 5.41 (m, 4H, H-3″ and H-3′′′); 4.55 (d, *J* = 6.6 Hz, 2H, H-1″); 4.51 (d, *J* = 6.6 Hz, 2H, H-1′′′). Spectroscopic data and physical properties of compound **3** were consistent with those reported in the literature [28].

*(2E)-1-{2,4-Bis[(2E)-but-2-en-1-yloxy]phenyl}-3-phenylprop-2-en-1-one* (**4**). Brown oil. Yield: 78.2%. Refractive index (n): 1.571. IR υ_max_ (KBr) cm^−1^: 2957 (C-H), 1636 (C=O), 1521 (C=C), 1245 (Ar-O-R), 1132 (C-O). ^1^H NMR (400.1 MHz, CDCl_3_): 7.84 (m, 1H, H-6′); 7.69 (d, *J* = 15.4 Hz, 1H, H-7); 7.64 (m, 2H, H-2 and H-6); 7.57 (1H, *J* = 15.5 Hz, H-8); 7.43 (m, 3H, H-3, H-4 and H-5); 6.51 (m, 2H, H-3′ and H-5′); 5.13 (s, 1H, H-3b″ H-3 b′′′); 5.04 (s, 1H, H-3α″ and H-3α′′′); 4.57 (d, *J* = 6.6 Hz, 2H, H-1″); 4.49 (d, *J* = 6.6 Hz, 2H, H-1′′′); 1.83 (s, 6H, H-4″ and H-4′′′). ^13^C NMR (100.6 MHz, CDCl_3_): 191.8 (C-9); 166.6 (C-2′); 165.4 (C-4′); 144.8 (C-7); 139.9 (C-2″ and C-2′′′); 134.8 (C-1); 131.2 (C-60); 130.6 (C-4); 129.0 (C-3 and C-5); 128.7 (C-2 and C-6); 122.4 (C-10); 120.4 (C-8); 113.4 (C-3″ and C-3′′′); 108.2 (C-5′); 102.0 (C-3′); 72.3 (C-1″); 71.9 (C-1′′′); 19.3 (C-4″ and C-4′′′). HRMS: M + H ion *m*/*z* 349.4347 (calculated for C_23_H_24_O_3_: 348.4348).

*(2E)-1-{2,4-Bis[(2-methylprop-2-en-1-yl)oxy]phenyl}-3-phenylprop-2-en-1-one* (**5**). Brown solid. Yield: 75.3%. m.p.: 108–110 °C. IR υ_max_ (KBr) cm^−1^: 2958 (C-H), 1635 (C=O), 1519 (C=C), 1243 (Ar-O-R), 1131 (C-O). ^1^H NMR (400.1 MHz, CDCl_3_): 7.84 (m, 1H, H-6′); 7.69 (d, *J* = 15.4 Hz, 1H, H-7); 7.64 (m, 2H, H-2 and H-6); 7.57 (1H, *J* = 15.5 Hz, H-8); 7.43 (m, 3H, H-3, H-4 and H-5); 6.49 (m, 2H, H-3′ and H-5′); 5.95 (m, 1H, H-2″ and H-2′′′); 5.75 (m, 1H, H-3″ and H-3′′′); 4.57 (d, *J* = 6.1 Hz, 2H, H-1″); 4.52 (d, *J* = 6.1 Hz, 2H, H-1′′′); 1.77 (s, 6H, H-4″ and H-4′′′). ^13^C NMR (100.6 MHz, CDCl_3_): 191.5 (C-9); 166.6 (C-2′); 165.4 (C-4′); 144.8 (C-7); 134.8 (C-1); 131.6 (C-2″ and C-2′′′); 131.1 (C-6′); 130.6 (C-4); 129.0 (C-3 and C-5); 128.5 (C-2 and C-6); 125.0 (C-1′); 120.4 (C-8); 114.1 (C-3″ and C-3′′′); 108.3 (C-5′); 101.8 (C-3′); 69.4 (C-1″); 68.9 (C-1′′′); 17.9 (C-4″ and C-4′′′). HRMS: M + H ion *m*/*z* 349.4441 (calculated for C_23_H_24_O_3_: 348.4448).

*(2E)-1-[2,4-Bis(but-3-en-1-yloxy)phenyl]-3-phenylprop-2-en-1-one* (**6**). Brown solid. Yield: 79.2%. m.p.: 95–97 °C. IR υ_max_ (KBr) cm^−1^: 2950 (C-H), 1636 (C=O), 1524 (C=C), 1248 (Ar-O-R), 1130 (C-O). ^1^H NMR (400.1 MHz, CDCl_3_): 7.84 (m, 1H, H-6′); 7.68 (d, *J* = 15.5 Hz, 1H, H-7); 7.66 (m, 2H, H-2 and H-6); 7.57 (1H, *J* = 15.5 Hz, H-8); 7.43 (m, 3H, H-3, H-4 and H-5); 6.49 (m, 2H, H-3′ and H-5′); 5.92 (m, 2H, H-3″ and H-3′′′); 5.19 (m, 4H, H-4″ and H-4′′′); 4.19 (d, *J* = 6.5 Hz, 2H, H-1″); 4.12 (d, *J* = 6.6 Hz, 2H, H-1′′′); 2.59 (m, 4H, H-2″ and H-2′′′). ^13^C NMR (100.6 MHz, CDCl_3_): 191.9 (C-9); 166.7 (C-4′); 165.4 (C-2′); 144.4 (C-7); 134.8 (C-1); 133.9 (C-3″ and C-3′′′); 131.2 (C-6′); 130.6 (C-4); 129.0 (C-3 and C-5); 128.5 (C-2 and C-6); 120.4 (C-8); 117.4 (C-4″ and C-4′′′); 114.1 (C-1′); 108.1 (C-5′); 101.6 (C-3′); 67.9 (C-1″ and C-1′′′); 33.4 (C-2″ and C-2′′′). HRMS: M + H ion *m*/*z* 349.4347 (calculated for C_23_H_24_O_3_: 348.4349).

*(2E)-1-{2,4-Bis[(3-methylbut-2-en-1-yl)oxy]phenyl}-3-phenylprop-2-en-1-one* (**7**). Brown solid. Yield: 80.0%. m.p.: 103–104 °C. IR υ_max_ (KBr) cm^−1^: 2958 (C-H), 1633 (C=O), 1517 (C=C), 1240 (Ar-O-R), 1130 (C-O). ^1^H NMR (400.1 MHz, CDCl_3_): 7.84 (m, 1H, H-6′); 7.69 (d, *J* = 15.6 Hz, 1H, H-7); 7.65 (m, 2H, H-2 and H-6); 7.56 (1H, *J* = 15.5 Hz, H-8); 7.43 (m, 3H, H-3, H-4 and H-5); 6.48 (m, 2H, H-3′ and H-5′); 5.87 (m, 2H, H-4″ and H-4′′′); 5.08 (m, 4H, H-5″ and H-5′′′); 4.07 (d, *J* = 6.4 Hz, 2H, H-1″); 4.01 (d, *J* = 6.3 Hz, 2H, H-1′′′); (m, 4H, H-1″ and H-1′′′); 2.27 (m, 4H, H-3″ and H-3′′′); 1.94 (m, 4H, H-2″ and H-2′′′); ^13^C NMR (100.6 MHz, CDCl_3_): 191.6 (C-9); 166.7 (C-4′); 165.8 (C-2′); 144.3 (C-7); 137.4 (C-4″ and C-4′′′); 134.8 (C-1); 131.2 (C-6′); 130.6 (C-4); 129.0 (C-3 and C-5); 128.5 (C-2 and C-6); 120.4 (C-8); 115.5 (C-5″ and C-5′′′); 114.0 (C-1′); 108.1 (C-5′); 101.6 (C-3′); 67.9 (C-1″ and C-1′′′); 30.0 (C-2″ and C-2′′′); 28.1 (C-3″ and C-3′′′). HRMS: M + H ion *m*/*z* 377.4881 (calculated for C_25_H_28_O_3_: 376.4890).

*(2E)-1-[2,4-Bis(pent-4-en-1-yloxy)phenyl]-3-phenylprop-2-en-1-one* (**8**). Yelow oil. Yield: 73.3%. Refractive index (n): 1.573. IR υ_max_ (KBr) cm^−1^: 2959 (C-H), 1633 (C=O), 1524 (C=C), 1245 (Ar-O-R), 1120 (C-O). ^1^H NMR (400.1 MHz, CDCl_3_): 7.84 (d, J =9.6 Hz, 1H, H-6′); 7.91 (d, *J* = 15.4 Hz, 1H, H-7); 7.30 (m, 2H, H-2 and H-6); 7.57 (1H, *J* = 15.5 Hz, H-8); 7.42 (m, 3H, H-3, H-4 and H-5); 6,51 (m, 2H, H-3′ and H-5′); 5.53 (m, 1H, H-2″); 5.49 (m, 1H, H-2′′′); 4.67 (d, *J* = 6.9 Hz, 2H, H-1″); 4.59 (d, *J* = 6.9 Hz, 2H, H-1′′′); 1.81 (s, 6H, H-4″ and H-4′′′); 1.79 (s, 6H, H-5″ and H-5′′′). ^13^C NMR (100.6 MHz, CDCl_3_): 190.5 (C-9); 166.7 (C-2′); 165.6 (C-4′); 144.3 (C-7); 139.2 (C-3′′′); 138.6 (C-3″); 134.9 (C-1); 131.2 (C-6′); 130.6 (C-4); 129.0 (C-3 and C-5); 128.5 (C-2 and C-6); 120.4 (C-8); 118.7 (C-2″); 114.0 (C-1′); 108.3 (C-5′); 101.7 (C-3′); 64.6 (C-1″); 64.1 (C-1′′′); 25.8 (C-5″ and C-5′′′); 18,2 (C-4″ and C-4′′′). HRMS: M+H ion *m*/*z* 377.4709 (C_25_H_28_O_3_: 376.4712).

*(2E)-1-(2,4-Bis{[(2E)-3,7-dimethylocta-2,6-dien-1-yl]oxy}phenyl)-3-phenylprop-2-en-1-one* (**9**). Brown oil. Yield: 69.8%. Refractive index (n): 1.553. IR υ_max_ (KBr) cm^−1^: 2940 and 2865 (C-H), 1633 (C=O), 1532 (C=C), 1241 (Ar-O-R), 1129 (C-O); ^1^H NMR (400.1 MHz, CDCl_3_): 7.91 (s, 1H, H-7); 7.84 (d, *J* = 9.6 Hz, 1H, H-6′); 7.65 (m, 2H, H-2 and H-6); 7.57 (1H, *J* = 15.5 Hz, H-8); 7.43 (m, 3H, H-3, H-4 and H-5); 6.50 (m, 2H, H-3′ and H-5′); 5.48 (m, 2H, H-2″ and H-2′′′); 5.09 (m, 2H, H-6″ and H-6′′′); 4.60 (d, *J* = 6.7 Hz, 2H, H-1″); 4.49 (d, *J* = 6.6 Hz, 2H, H-1′′′); 2.12 (m, 4H, H-4″, H-4′′′, H-5″ and H-5′′′), 1.75 (s, 6H, H-8″ and H-8′′′); 1.68 (s, 6H, H-9″ and H-9′′′), 1.61 (s, 6H, H-10″ and H-10′′′). ^13^C NMR (100.6 MHz, CDCl_3_): 190.8 (C-9); 166.7 (C-2′); 166.0 (C-4′); 144.3 (C-7); 141.2 (C-3″ and C-3′′′); 134.9 (C-1); 132.1 (C-6′); 131.2 (C-7″ and C-7′′′); 130.6 (C-4); 129.0 (C-3 and C-5); 128.5 (C-2 and C-6); 123.7 (C-6″ and C-6′′′); 120.4 (C-8); 119.6 (C-2″); 118.5 (C-2′′′); 114.0 (C-1′); 108.3 (C-5′); 101.8 (C-3′); 66.4 (C-1″); 65.3 (C-1′′′); 39.5 (C-4″ and C-4′′′); 26.3 (C-5″ and C-5′′′); 25.6 (C-10″ and C-10′′′); 17.7 (C-9″ and C-9′′′); 16.7 (C-8″ and C-8′′′). HRMS: M + H ion *m*/*z* 513.7119 (calculated for C_35_H_44_O_3_: 512.7130).

*(2E)-1-(2,4-Bis{[(2E,6E)-3,7,11-trimethyldodeca-2,6,10-trien-1-yl]oxy}phenyl)-3-phenylprop-2-en-1-one* (**10**). Brown oil. Yield: 60.4%. Refractive index (n): 1.547. IR υ_max_ (KBr) cm^−1^: 2955 (C-H), 1632 (C=O), 1543 (C=C), 1243 (Ar-O-R), 1140 (C-O). ^1^H NMR (400.1 MHz, CDCl_3_): 7.84 (d, *J* = 8.6 Hz, 1H, H-6′); 7.69 (s, 1H, H-7); 7.65 (m, 2H, H-2 and H-6); 7.58 (1H, *J* = 15.5 Hz, H-8); 7.35 (m, 3H, H-3, H-4 and H-5); 6.57 (m, 1H, H-5′); 6.52 (m, 1H, H-3′); 5.55 (m, 1H, H-2′′′); 5.50 (m, 1H, H-2″); 5.09 (m, 4H, H-6″, H-6′′′, H-10″ and H-10′′′); 4.60 (m, 4H, H-1″ and H-1′′′); 2.07 (m, 8H, H-4″, H-4′′′, H-5″ and H-5′′′), 1.97 (m, 8H, H-8″, H-8′′′. H-9″ and H-9′′′), 1.76 (s, 3H, H-12″); 1.74 (s, 3H, H-12′′′); 1.68 (s, 6H, H-15″ and H-15′′′); 1.59 (s, 12H, H-13″, H-13′′′, H-14″ and H-14′′′). ^13^C NMR (100.6 MHz, CDCl_3_): 190.5 (C-9); 163.7 (C-2′); 160.1 (C-4′); 142.0 (C-7); 142.0 (C-3″ and C-3′′′); 135.8 (C-7″ and C-7′′′); 135.6 (C-1); 133.1 (C-6′); 131.4 (C-11″ and C-11′′′); 129.7 (C-4); 128.7 (C-3 and C-5); 128.2 (C-2 and C-6); 124.3 (C-6″ and C-6′′′); 123.6 (C-10″ and C-10′′′); 122.1 (C-8); 118.9 (C-2″ and C-2′′′); 118.7 (C-1′); 106.1 (C-5′); 100.3 (C-3′); 65.6 (C-1″ and C-1′′′); 39.7 (C-4″ and C-4′′′); 39.6 (C-8″ and C-8′′′); 26.2 (C-5″, C-5′′′, C-9″ and C-9″’); 25.7 (C-15″ and C-15′′′); 17.7 (C-14″ and C-14′′′); 16.7 (C-12″ and C-12′′′); 16.0 (C-13″ and C-13′′′). HRMS: M + H ion *m*/*z* 649.9563 (calculated for C_45_H_60_O_3_: 648.9561).

### 3.3. *Saprolegnia australis* Strain

The strains of *S. australis* were isolated from *Salmo salar* carp eggs [12].

### 3.4. Anti-Saprolegnia Activity

#### 3.4.1. Minimum Inhibitory Concentration

The minimum inhibitory concentrations (MIC) were assessed by the procedure previously described in reference [12]. Bronopol and fluconazole were used as positive controls.

#### 3.4.2. Minimum Oomycidal Concentrations

The minimum oomycidal concentrations (MOC) were assessed by the procedure previously described in reference [12]. Bronopol and fluconazole were used as positive controls.

#### 3.4.3. Cellular Leakage

The percentage of cellular leakage was assessed by the procedure previously described in reference [12]. Sodium dodecyl sulfate was used as a reference compound.

### 3.5. Statistical Analysis

Determinations of MIC and MOC were performed in triplicate and the results expressed as mean values ± SD. The results were analyzed using the standard method [12].

### 3.6. Computational Details

The solvent influence in the reaction of compound **1** and solvent (acetone and DMF) was optimized using DFT-B3LYP-6-31G incorporated into Gaussian 03 program [29], obtained under different steric-electronic parameters. Each one of the optimized geometries was verified by absence of imaginary frequency. After optimization, the electrostatic potential map was plotted (ESP-plot).

On the other hand, the dialkoxychalcones presented in Scheme 1 were subjected to the following computational treatment. Full unconstrained geometry optimizations of these compounds were carried out using Gaussian 03 program [29] using DFT-B3LYP-6-31G in gas and solvent (water) phases. Optimized geometries were verified by absence of imaginary frequency in their potential energy surface. The reactivity descriptors Highest Orbital Molecular Occupied (HOMO), Lowest Orbital Molecular Unoccupied (LUMO), difference between LUMO and HOMO (ΔLH), electronic chemical potential (µ), Hardness (η), Softness (S), and Electrophilicity Global Index (ω) were calculated with the following equations:

μ = (E_LUMO_ + E_HOMO_)/2 (3)

η = (E_LUMO_ − E_HOMO_)/2 (4)

S = 1/2η(5)

ω = μ^2^/2η (6)

As well as natural bonding orbital charge of chalcone skeleton C1, C2, C3, C4, C5, C6, C1′, C2′, C3′, C4′, C5′, C6′, Cα, Cβ, and CO (NBO-Charge). In addition, topological and steric descriptors, such as molecular weight (MW), the logarithm of the partition coefficient between n-octanol and water (CLogP), molar refractivity (MR), molecular area (MA), molecular volume (MV), hydrogen bond acceptor (HBA), hydrogen bonding donor (HBD), Balaban index (BI), molecular topological index (MTI), rotatable bonds (RB), topological diameter (TD), and Wiener index (WI), were obtained from Chem-Draw software.

### 3.7. Structure-Activity Relationship

For this analysis, multiple linear regressions were used to check the influence of chemical features and anti-oomycete activity. The analysis was performed selecting different descriptors, such as MW, CLogP, MR, MA, MV, HBA, HBD, BI, MTI, RB, topological diameter TD, and WI. In addition, HOMO, LUMO, ΔLH, µ, η, S, and ω NBO-Charge on C1, C2, C3, C4, C5, C6, C1′, C2′, C3′, C4′, C5′, C6′, Cα, Cβ, and CO were used in gas and solvent phases. Model optimization was carried out by Statistica 7.0 Package Software.

### 3.8. Cross-Validation QSRR Model

Cross-validation of QSRR model was carried out using the Golbraikh method [30]. Acceptable q^2^ values are equal or greater than 0.5. q^2^ values are obtained by following formula:

q^2^ = 1 − (∑(y_obs_ − y_calc_)^2^)/(∑(y_obs_ − y_ave_)^2^), (7)

where y_obs_ is pMIC observed, y_calc_ is pMIC calculated by QSAR model gas or solvent phase, and y_ave_ is pMIC average of all compounds in the training set.

## 4. Conclusions

In conclusion, a series of chalcone derivatives **2**–**10** with typical chalcone cores have been successfully synthesized and found to display strong to weak oomycidal agents against *Saprolegnia australis* strains. Compound **8** with two allyloxy chains (C_5_) was found to exhibit a more potent anti-oomycete activity than the other alkoxy chalcone derivatives and the standard drugs, bronopol and fluconazole, used in the assays, revealing the structural framework to have application advantage associated with strong anti-oomycete effect elicited on *Saprolegnia australis* strains. Additionally, a study of structure–activity relationship was carried out using the calculations of all compounds in gas phase and solvent phase (water) using various topological, steric, and quantum descriptors in conjunction with multivariate regression models, obtaining that the model in gas phase possesses better statistical values than the model in solvent phase (r^2^_gas_ = 0.945, SD_gas_ = 0.154, r^2^_solv_ = 0.881, SD_solv_ = 0.201). In this context, the global electrophilicity index (ω) is the most important descriptor for explaining anti-oomycete activity.

## Figures and Tables

**Table 1:** In vitro anti-oomycete activities values ^a^ of synthesized compounds against *S. australis*.

Compound	MIC (µg·mL^−1^)	MOC (µg·mL^−1^)	Damage (%) ^b^
**1**	6.25	12.5	100
**2**	150	175	5
**3**	200	>200	0
**4**	75	100	74
**5**	100	125	37
**6**	>200	>200	0
**7**	>200	>200	0
**8**	50	50	81
**9**	>200	>200	0
**10**	150	175	20
Bronopol	>200	>200	30
Fluconazole	150	175	Nd
SDS	Nd	Nd	100

Nd: Not determined; ^a^ Each value represents the mean ± SD of three experiments, performed in triplicate; ^b^ Damage produced by compounds **1**–**8** compared to the damaged produced by Sodium Dodecyl Sulfate (SDS). SDS was utilized at a final concentration of 2% that produced 100% of cell lysis. The assay was performed in duplicates.

## Supplementary Materials

The following are available online. Table S1: Descriptors obtained in gas phase and used for structure–activity relationship, Table S2: Descriptors obtained in solvent phase and used for structure–activity relationship.
