# Supplementary material for: Structure-Activity Relationship of Dialkoxychalcones to Combat Fish Pathogen Saprolegnia australis
Source: Molecules. 2018 Jun 7;23(6):1377. doi: 10.3390/molecules23061377 (PMC6100462; doi:10.3390/molecules23061377)
Supplement: Supplementary file 1 [file molecules-23-01377-s001.zip › molecules-307471-SI.pdf]

SUPPLEMENTARY MATERIAL

Structure-Activity Relationship of Dialkoxychalcones to Combat Fish Pathogen *Saprolegnia australis*

Iván Montenegro [1], Ociel Muñoz [2], Joan Villena [3], Enrique Werner [4], Marco Mellado [5], Ingrid Ramírez [6], Nelson Caro [7], Susana Flores [8] and Alejandro Madrid [8,*]

[1] Escuela de Obstetricia y Puericultura, Facultad de medicina, Campus de la Salud, Universidad de Valparaíso, Angamos 655, Reñaca, Viña del Mar 2520000, Chile; ivan.montenegro@uv.cl
[2] Institute of Food Science and Technology, University Austral of Chile, Valdivia 5090000, Chile; ocielmunoz@uach.cl
[3] Centro de Investigaciones Biomédicas (CIB), Escuela de Medicina, Universidad de Valparaíso, Av. Hontaneda N° 2664, Valparaíso 2340000, Chile; juan.villena@uv.cl
[4] Departamento De Ciencias Básicas, Campus Fernando May Universidad del Biobío. Avda. Andrés Bello s/n casilla 447, Chillán 3780000, Chile; ewerner@ubiobio.cl
[5] Instituto de Química, Facultad de Ciencias, Pontificia Universidad Católica de Valparaíso, Av. Universidad #330, Curauma, Valparaíso 2340000, Chile; marco.mellado@pucv.cl
[6] Centro de Biotecnología "Dr. Daniel Alkalay Lowitt", Universidad Técnica Federico Santa María, Avda. España 1680, Valparaíso 2340000, Chile; ingrid.ramirez@usm.cl
[7] Centro de Investigación Australbiotech, Universidad Santo Tomás, Avda. Ejército 146, Santiago 8320000, Chile; ncaro@australbiotech.cl
[8] Departamento de Química, Facultad de Ciencias Naturales y Exactas, Universidad de Playa Ancha, Avda. Leopoldo Carvallo 270, Playa Ancha, Valparaíso 2340000, Chile; s.flores.gonzalez@gmail.com
* Correspondence: alejandro.madrid@upla.cl; Tel.: +56-032-250-0526

Experimental and calculated properties of compounds 1, 2, 3, 5, 8 and 10

1. **Table S1** 2
2. **Table S2** 3

Table S1. Descriptors obtained in gas phase and used for structure-activity relationship

Compound	$LUMO^{-1}$	$Log_{10}\omega$	pIC_{50} observed	pIC_{50} Calculated	Residual
**1**	-11.471	-0.753	4.584	4.584	0.000
**2**	-14.478	-0.859	3.252	3.200	0.052
**3**	-14.575	-0.862	3.204	3.272	-0.068
**5**	-15.088	-0.876	3.542	3.705	-0.164
**8**	-15.078	-0.876	3.876	3.685	0.192
**10**	-15.044	-0.875	3.636	3.649	-0.013

Table S2. Descriptors obtained in solvent phase and used for structure-activity relationship

Compound	$HOMO^{-1}$	η^{-1}	pIC_{50} observed	pIC_{50} Calculated	Residual
**1**	-4.325	13.458	4.584	4.511	0.073
**2**	-4.412	13.822	3.252	3.495	-0.242
**4**	-4.406	13.791	3.667	3.686	-0.020
**5**	-4.388	13.729	3.542	3.323	0.218
**8**	-4.443	13.934	3.876	3.958	-0.082
**10**	-4.451	13.909	3.636	3.584	0.053
